# Stability of citrate and calcium dosing in citrate renal replacement therapy

**DOI:** 10.1186/2197-425X-3-S1-A63

**Published:** 2015-10-01

**Authors:** F Sadaat, E Walter

**Affiliations:** Royal Surrey County Hospital, Intensive Care Unit, Guildford, United Kingdom

## Introduction

Citrate is the recommended anticoagulant for continuous renal replacement therapy (RRT)[1], and is thought to confer numerous benefits, including more continuous hours of filtration, fewer total circuits used, less overall cost and maybe improved patient and kidney survival when compared with heparin anticoagulation[2]. Citrate is run pre filter, calcium is infused post filter, the rates of both determined by the filter and patient ionised calcium level respectively. This is monitored by blood tests, which must be sampled frequently. Filter and patient ionised calcium levels, citrate and calcium infusion rates, are recorded as part of clinical practice at RSCH. The target plasma ionised calcium concentration is 1.0 - 1.2mM, the target filter ionised calcium concentration is 0.25 - 0.5mM.

## Objectives

There are currently little data available on how often the rates of citrate and calcium infusion are changed, and consequently some concern amongst nursing staff regarding the frequency of blood sampling and associated workload.

## Methods

Observational data were recorded on the time intervals between the changes in the calcium chloride, and citrate infusion rate. Data were collected prospectively for the first five patients, up to and including the first 15 days following citrate introduction.

## Results

**Figure 1:**
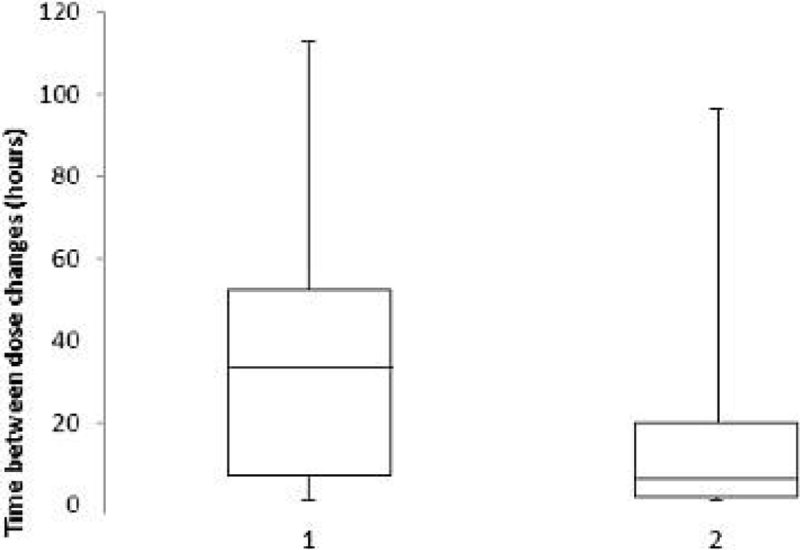
**Box Whisker Plot to show hours between dose changes of citrate (1) and calcium (2). Boxes represent the interquartile range and median, whiskers represent the minimum and maximum time interval (hours).**

Data from the first five patients filtered with citrate are presented, representing a total of 736 hours of RRT. The median length of time of citrate infusion between dose changes was 32 hours (interquartile range 7.0 - 52.2 hours), with the longest period 112.7 hours. The median length of time of calcium infusion between dose changes was 6.3 hours (interquartile range 2.0 - 20.0 hours) with the longest period 96.5 hours (Figure 1).

## Conclusions

When citrate anticoagulation is used for renal replacement therapy, there are long periods when neither citrate nor calcium infusion rates require altering. These long periods often follow a small cluster of dose changes, as a 'steady state' is achieved. As a result, blood samples are required less frequently, which in turn, contributes to a lighter workload for nursing staff and lower operational costs.
